# Supplementary material for: Content-rich biological network constructed by mining PubMed abstracts
Source: BMC Bioinformatics. 2004 Oct 8;5:147. doi: 10.1186/1471-2105-5-147 (PMC528731; doi:10.1186/1471-2105-5-147)
Supplement: Additional File 2 — The original results of the above study (non-essential files are deleted to keep the file size under the limit set by BMC bioinformatics). [file 1471-2105-5-147-S2.bz2 › chilibotAdditionalFile2/dip10/258ID2569363E1022/html/CDC13_CDC2.html]

 


 **CDC13** and **CDC2** 
  
Found 69 abstracts in PubMed, retrieved 10.  
 

 What does Google say? 
 PDF only 
| .edu only 

---

**Interactive relationship** (e.g. stimulation, inhibition, etc)

**Neutral relationship**- Nuclear  **cdc2**  YFP fluorescence oscillates with that of  **cdc13**  YFP cyclin.  Ref: 11683390 J Cell Sci, 2001

**Non-interactive relationship** (e.g. studied together, co-existance, homology, etc.)

- We show that in fission yeast the mitotic B type cyclin  **Cdc13**   **Cdc2**  kinase associates with replication origins in vivo.  Ref: 12419251 Cell, 2002
- An mfr1 null mutant completes meiosis II but remains with high levels of  **cdc13**  and  **cdc2**  kinase activity and has considerably delayed spore formation.  Ref: 11493649 J Cell Sci, 2001
- To investigate the mechanism coupling growth protein synthesis with cell division, we examined the relationship between the tyrosine kinase Wee1 that inhibits  **Cdc2**   **Cdc13**  mitosis inducing kinase by phosphorylating it, and protein synthesis inhibition in fission yeast.  Ref: 11119724 FEBS Lett, 2000
- **Cdc2**  YFP and  **cdc13**  YFP are highly enriched on the spindle pole body of cells in late G2 or arrested at S phase.  Ref: 11683390 J Cell Sci, 2001
- This mitotic induction operates, at least in part, through activation of the normal G 2 M CDK  [ **CDC2** ]  complex  **Cdc13**   **Cdc2** .  Ref: 12093738 EMBO J, 2002
- If cdc13p cannot be recognized by the anaphase promoting complex,  **cdc2**  YFP and  **cdc13**  YFP remain associated with the spindle.  Ref: 11683390 J Cell Sci, 2001
